# Interactions Between Light Intensity and Phosphorus Nutrition Affect the P Uptake Capacity of Maize and Soybean Seedling in a Low Light Intensity Area

**DOI:** 10.3389/fpls.2019.00183

**Published:** 2019-02-19

**Authors:** Tao Zhou, Li Wang, Shuxian Li, Yang Gao, Yongli Du, Li Zhao, Weiguo Liu, Wenyu Yang

**Affiliations:** ^1^College of Agronomy, Sichuan Agricultural University, Chengdu, China; ^2^Sichuan Coalfield Geological Bureau, Chengdu, China; ^3^Key Laboratory of Crop Ecophysiology and Farming System in Southwest, Ministry of Agriculture, Chengdu, China

**Keywords:** photosynthesis, P uptake capacity, root growth, sucrose, maize, soybean, carboxylates

## Abstract

To capture more nutrients, root systems of maize (*Zea mays* L.) and soybean (*Glycine max* L.) exhibit morphological and physiological plasticity to a localized supply of phosphorus (P). However, the mechanisms of the interaction between light intensity and P affecting root morphological and physiological alterations remain unclear. In the present study, the regulation of P uptake capacity of maize and soybean by light intensity and localized P supply was investigated in a low solar radiation area. The plants were grown under continual and disrupted light conditions with homogeneous and heterogeneous P supply. Light capture of maize and soybean increased under the disrupted light condition. Plant dry weight and P uptake were increased by more light capture, particularly when plants were grown in soil with heterogeneous P supply. Similarly, both localized P supply and disrupted light treatment increased the production of fine roots and specific root length in maize. Both homogeneous P supply and disrupted light treatment increased the malate and citrate exudation in the root of soybean. Across all of the experimental treatments, high root morphological plasticity of maize and root physiological plasticity of soybean were associated with lower P concentrations in leaves and greater sucrose concentrations in roots. It is suggested that the carbon (C), exceeded shoot growth capabilities and was transferred to roots as sucrose, which may serve as both a nutritional signal and a C-substrate for root morphological and physiological changes.

## Introduction

Light intensity plays an important role in determining the performance of individuals in natural communities and the growth and productivity of crops in agroecological systems. In the Sichuan Basin located upstream on the Yangtze River, the range of total solar radiation is 3350–4190 MJ m^-2^ per year and below the annual average of 5900 MJ m^-2^ for China in general. Due to low solar radiation, local crops are sown at low density (maize at 6.0 × 10^4^ plant ha^-1^, soybean at 1.2 × 10^5^ plant ha^-1^, rice at 1.3 × 10^5^ plant ha^-1^, and wheat at 2.4 × 10^7^ plant ha^-1^) to circumvent the self-shading within the canopy. Shading is ubiquitous, and all plants are shaded to some degree during their lifetime (Valladares and Niinemets, 2008). Practically, crops capture more light by changing the planting pattern from equal row width distance to narrow-wide row distances rather than decreasing the planting density, as shown in Supplementary Figure S1 (Liu and Song, 2012). The availability of solar radiation in the narrow row decreases with decreasing row width. However, the light capture of crops in the narrow-wide planting pattern is much higher than that of crops in the equal-width row planting pattern. This might be mostly attributed to the light interception in wide rows (Wang et al., 2017). In addition to the amelioration of light conditions in the wide spacing of the narrow-wide row distance planting pattern, plant phenotypic plasticity may markedly contribute to capture due to minimizing the self-shading effect (Zhu et al., 2015, 2016). Additionally, the leaves of crops maintain a longer green period in the narrow-wide row distance planting pattern than that in the equal-width row distance planting pattern, resulting in leaf area, subsequently, and light capture increased.

It is widely acknowledged that, of the applied fertilizer P each season, crop uptake is only 10–25% (Johnston et al., 2014), as P is strongly bound to soil particles (Hinsinger, 2001; Shen et al., 2011). Roots in soils with low availability and heterogeneous distribution of P show high plasticity to increase the capacity of P acquisition, including root morphological and physiological strategies (Lambers et al., 2006; Shen et al., 2011). For example, cluster-root formation and citrate exudation of *Lupinus albus* were induced by low soil P availability and inhibited by increased P supply (Shen et al., 2003; Li et al., 2008). In many species, including maize and wheat, the heterogeneous distribution of P affects root growth and distribution (Jing et al., 2010; Li L. et al., 2014). The proliferation of roots increases in local nutrient-rich zones because plants stimulate root growth and alter root distribution in response to nutrient-rich zones (Hodge, 2004). However, not all crop species (e.g., Faba bean) show a significant root growth response to localized P supply (Li H.B. et al., 2014; Zhang et al., 2016). Similarly, some species show positive root physiological responses to localized nutrient enrichment (Jackson et al., 1990). Nutrients localized in the soil not only alter root morphology and physiology but also are used as an effective management strategy to determine root distribution in the soil profile. Greater root distribution in wide rows than that in narrow rows in response to localized P supply reduces root competition and increases the acquisition of P from soil (Li Y. et al., 2016; Li H.B. et al., 2016). Hence, the plasticity of root morphology, physiology, and distribution in foraging P is also determined by soil environmental conditions.

Many studies have investigated the contributions of changes in root morphology and physiology for P acquisition in soil environments with low P availability (Rengel and Marschner, 2005; Wang et al., 2009, 2010; Yuan and Chen, 2012; Zhang et al., 2012; Fernandez and Rubio, 2015). Less attention has been paid to understanding how the light aboveground influences root morphology and physiology. Studies indicate that photosynthate is preferentially distributed to shoots during leaf extension to increase the interception of light and decrease the ratios of root to shoot biomass when plants are grown under low light intensity conditions (Gommers et al., 2013; Gundel et al., 2014). Simultaneously, total root length decreased when plants are grown in low light intensities (Samuel and Matthew, 2004; Wissuwa et al., 2005; Sparkes et al., 2008). Light is not only involved in the synthesis and transportation of photosynthate, but also as signal direct regulates root morphology. Such as far-red light detection in the shoot of Arabidopsis regulates lateral root growth through the HY5 transcription factor (Van Gelderen et al., 2018a,b). Root morphology is also altered by shoot P concentration and root sucrose concentration, and the tissue P and sucrose concentration affected by light intensity. High light intensity increases cluster-root formation of white lupine, which is associated with an increase in root sucrose concentration under P-deficient conditions (Cheng et al., 2014). Exogenous supply of sucrose also stimulates the formation of cluster roots, even in P-sufficient conditions (Zhou et al., 2008). High light intensity decreases shoot P concentration, which subsequently increases the total root length, specific root length, and production of fine roots (Cheng et al., 2014; Wen et al., 2017). Root physiology is also affected by light intensity. For example, the expression of the gene *LaPEPC3* (which initiates citrate synthesis) and citrate exudation of white lupine increased with increasing light intensity, independent of the response to changing P supply (Cheng et al., 2014).

In response to low soil P conditions, maize altered root morphology rather than root physiology (Lyu et al., 2016; Wen et al., 2017), whereas soybean positively altered root morphology and root physiology in response to low P soil (Lyu et al., 2016). However, very few studies explore the effect of light intensity aboveground on root morphology and physiology, particularly for maize and soybean, which are widely cultivated crops globally, including in the typically low light intensity areas of southwest China. The primary purpose of this study was to investigate the interactions between heterogeneous shoot light intensity and localized P supply affecting root morphology and physiology associated with an increased ability of maize and soybean P acquisition. Specifically, the hypotheses were that (i), increasing light capture increases photosynthesis and shoot growth rate, which exceeds the P supply ability of roots to leaf, causing growth-induced P starvation in the shoot that produces a systemic signal to induce root proliferation and carboxylate exudation. Furthermore (ii), carbon accumulations in excess of what is required for shoot growth capabilities are transferred to roots as sucrose, which serves as both a nutritional signal and a carbon-substrate for root morphological and physiological changes.

## Materials and Methods

### Experimental Setup

**FIGURE 1:**
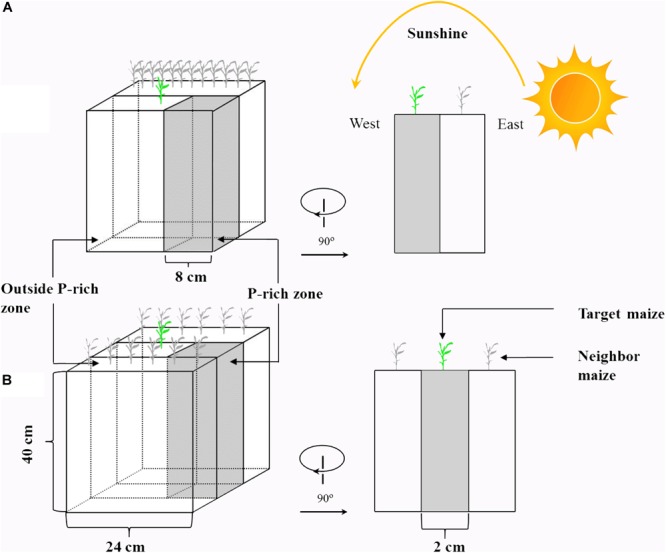
A schematic diagram of the rhizo-box. Shown are: disrupted light condition treatment **(A)** and continual light condition treatment **(B)**. The design of this experiment in maize and soybean was the same, but here only shown for maize to avoid redundancy.

Maize (*Zea mays* L., cv. CD418) and soybean (*Glycine max* L., cv. ND12) were cultivated to investigate their P uptake capacities in response to heterogeneity in light intensity and P in soil. A rhizo-box (24 × 2 × 40 cm, Figure 1) experiment with two light condition treatments (continual and disrupted light conditions) and two P supply treatments was conducted, with three replicates of each treatment. To change the light interception of the experimental plants, two PVC-rhizo-boxes were linked together; the target plants were grown in the one box, and 12 plants of the same species as neighboring for preventing light transmission were grown in the other box. The light interception of the target plants was mostly from the other side with nothing impeding the light (disrupted light condition) (Figure 1A). This treatment was designed to simulate plant growth maximally under disrupted light conditions, which simulated conditions as the “narrow-wide row distance planting pattern” in the field (Supplementary Figure S1). The continual light treatment was three PVC-rhizo-boxes linked together, with the target plants grown in the middle and the left and right boxes with six plants of the same species, respectively. The target plants intercepted light from the interstices among the neighbor plants (continual light condition) (Figure 1B). This treatment was designed to maximally simulate the target plant growth under a continual light condition as the “equal-width row distance planting pattern” in the field. The rhizo-boxes were placed to face to the east and the continual and disrupted light environment in the two treatments were induced by the sun trajectory from east to west (Figure 1).

The PVC-rhizo-boxes contained irrigation holes, a viscose fleece for moisture distribution, white plastic foil for a soil covering and a Perspex front lid with screws. All rhizo-boxes were filled with 3 kg of air-dried soil. Phosphorus was supplied as KH_2_PO_4_ in a homogeneous or heterogeneous pattern. For the heterogeneous P treatment, an 8-cm P-rich layer (1000 g of soil) containing 200 mg of P (200 mg P kg^-1^ soil) was manually mixed and placed at the right of the rhizo-box as the P-rich zone, and the remaining soil without P addition was the background soil (2000 g of soil, outside the P-rich zone) (Figure 1). For the homogeneous P treatment, the same total P (200 mg of P) was spread evenly throughout the soil (66 mg P kg^-1^ soil).

The soil was collected from the Renshou experimental station in Sichuan, China, air-dried and passed through a 2-mm sieve. Soil properties were as follow: Olsen-P 3.3 mg kg^-1^, organic matter 8.7 g kg^-1^, total N 0.3 g kg^-1^, available K 85 mg kg^-1^, available N 7.5 mg kg^-1^ and pH 6.7. To ensure that the nutrient supply was adequate for plant growth, soil was fertilized with basal nutrients at the following rates (mg per box): Ca(NO_3_)_2_⋅4H_2_O, 3374; K_2_SO_4_, 400; MgSO_4_⋅7H_2_O, 130; Fe-EDTA, 17.56; MnSO_4_⋅H_2_O, 20; ZnSO_4_⋅7H_2_O, 30; CuSO_4_⋅5H_2_O, 6; H_3_BO_3_, 2; and Na_2_MoO_4_⋅5H_2_O, 0.5.

Maize and soybean seeds were surface-sterilized in 30% v/v H_2_O_2_ for 20 min and washed with deionized water before planting. Before planting, all rhizo-boxes were irrigated through the irrigation holes. After 4 days of growth (when the plants emerged from the soil), the rhizo-boxes were irrigated every day until harvest. The maize and soybean were grown from July 12 to August 20, 2017.

**FIGURE 2:**
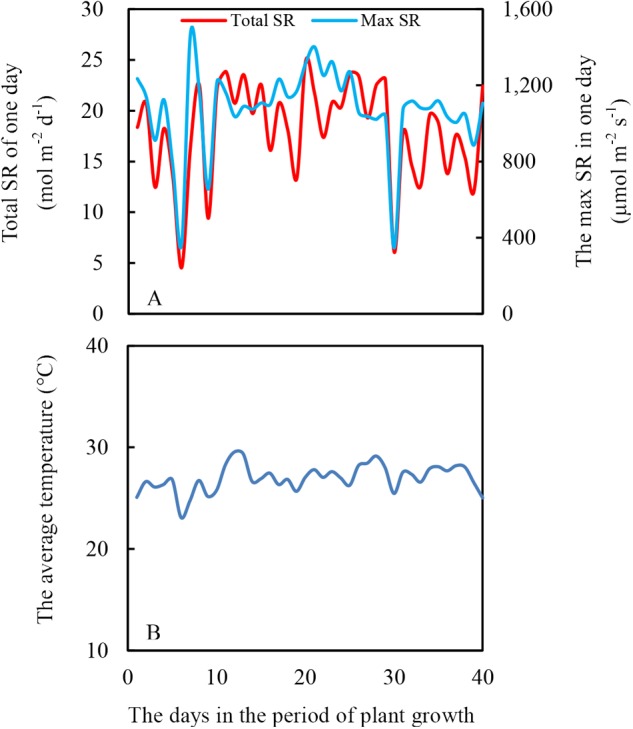
Daily total solar radiation (SR) **(A)** and average air temperature **(B)** during soybean and maize growth period, from July 12th to August 20th in 2017.

The experiment was conducted in a greenhouse at Sichuan Agricultural University, Chengdu (Latitude: 30°42′ N, Longitude: 103°51′ E). The height of the greenhouse was 6 m, and the top of the greenhouse was covered with thin and transparent plastic to prevent rainfall. Additionally, no plastic was vertically around the greenhouse to maintain the same air temperature inside to outside the greenhouse. The light intensity and air temperature are shown in Figure 2.

### Plant Harvest and Measurements of Root Morphology

After 40 days of growth, the plants were harvested. Shoots were oven-dried at 105°C for 30 min and at 80°C for 3 days for assays of dry weight and concentrations of P, starch, and sucrose. In the heterogeneous P treatment, roots were separated to the P-rich zone and outside the P-rich zone. In the homogeneous P treatment, the entire root systems were removed from the rhizo-boxes. Roots were washed with deionized water, and then WinRHIZO (WinRHIZO Pro2004, Canada) was used to measure root length and root diameter. Fine roots were defined as roots with a diameter less than 2 mm (Jing et al., 2010). After the determination of root morphology, roots were oven-dried at 105°C for 30 min and at 80°C for 3 days for assays of dry weight and concentrations of P and sucrose.

### Determination of Solar Radiation Interception Rate, Net Photosynthetic Rate, and Concentrations of P, Starch, and Sucrose

Solar radiation interception of plants was measured on all fully expanded leaves using OptoLeaf (Long-term measurement type, Japan) for a week (Kawamura et al., 2005). Net photosynthetic rate (Pn) was measured on the youngest fully expanded leaf using a Li6400 photosynthesis system (Li-COR, Lincoln, NE, United States). Measurements were performed between 10:00 a.m. and 12:00 p.m. on sunny days.

The P concentration in leaf, stem, and root was determined. The material was ground to pass through a 0.149-mm mesh sieve and a 0.3-g sample was wet-digested with concentrated H_2_SO_4_ and H_2_O_2_ (30%), and the P was determined by the vanadomolybdate method (Page, 1982).

The extracts of leaves and roots were analyzed after extraction in 80% ethanol (v/v). Sucrose concentration was measured directly in the extract, using resorcinol as the color reagent (Shi et al., 2016). The leaf sample residue after ethanol extraction was washed several times and used for starch analysis following the method of Setter et al. (1994).

### Collection and Analysis of Rhizosphere Soil Carboxylates

The soil adhering to the roots was defined as rhizosphere soil. The rhizosphere soil carboxylates were collected following the method of Zhang et al. (2015). Specifically, following removal from the rhizo-boxes, roots were transferred to a tube containing 50 ml of 0.2 μM CaCl_2_ and were gently shaken to dislodge the rhizosphere soil, followed by shaking for 5–10 s to create homogeneous suspensions. A 10 ml of the suspensions was freeze-dried at -80°C. Then the residue white powder was dissolved with 1 ml of deionized water for carboxylate analysis by high-performance liquid chromatography (HPLC). The HPLC analysis method was according to a previous report (Zhou et al., 2016). The static phase was a 250 × 4.6 mm C18 column (Hypsil, Dalian, China). The mobile phase was 0.5% KH_2_PO_4_ and 0.5 mM tetrabutylammonium hydrogen sulfate (pH 2.0) with a flow rate of 1 ml min^-1^ at 28°C, and the detection wavelength was 220 nm.

### Statistical Analyses

Data from the three replicates were sorted by the Excel (Microsoft) software packages. Analysis of variance (ANOVA) was conducted using the 19.0 statistical software packages (SPSS Institute Inc., United States). Significant differences among means were separated according to LSD at the level of *p* ≤ 0.05. Plant growth, Pn, and root length were subjected to one-way ANOVA to assess the effects of light interception and heterogeneous/homogeneous P supply in this study.

## Results

### Plant Biomass and Root Development

**FIGURE 3:**
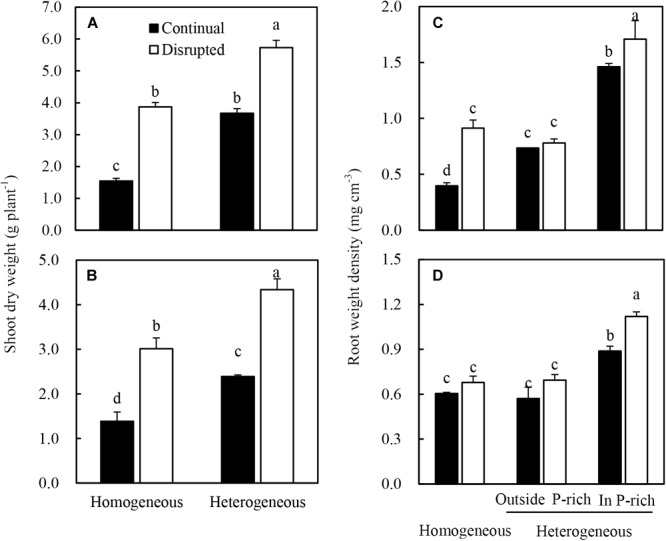
Effect of continual vs. disrupted light condition and homogeneous vs. heterogeneous phosphorus (P) supply on plants shoot dry weight (**A**: maize, **B**: soybean), and on root weight density (**C**: maize, **D**: soybean). Plants were grown in rhizo-box at two P supply treatments (homogeneous = homogeneous P supply, heterogeneous = heterogeneous P supply) and two light conditions (continual = continual light condition, disrupted = disrupted light condition). Data are average of three replicates and bars represent standard errors. Data with different letters are significantly different (*p* < 0.05).

The shoot dry weight of maize significantly increased under disrupted light conditions compared with continual light conditions irrespective of P supply (Figure 3A). Root dry weight density of maize increased when the light intensity changed from continual to disrupted in plant supplied with sufficient P (in the P-rich zone), but was not affected by the light condition when the root was grown with low P supply (outside the P-rich zone) (Figure 3C).

The shoot dry weight (Figure 3B) and root dry weight density (Figure 3D) of soybean generally followed similar tendencies to that of maize in the light and P treatments. However, root dry weight density of soybean was only significantly enhanced under disrupted light in the P-rich zone (Figure 3D).

**FIGURE 4:**
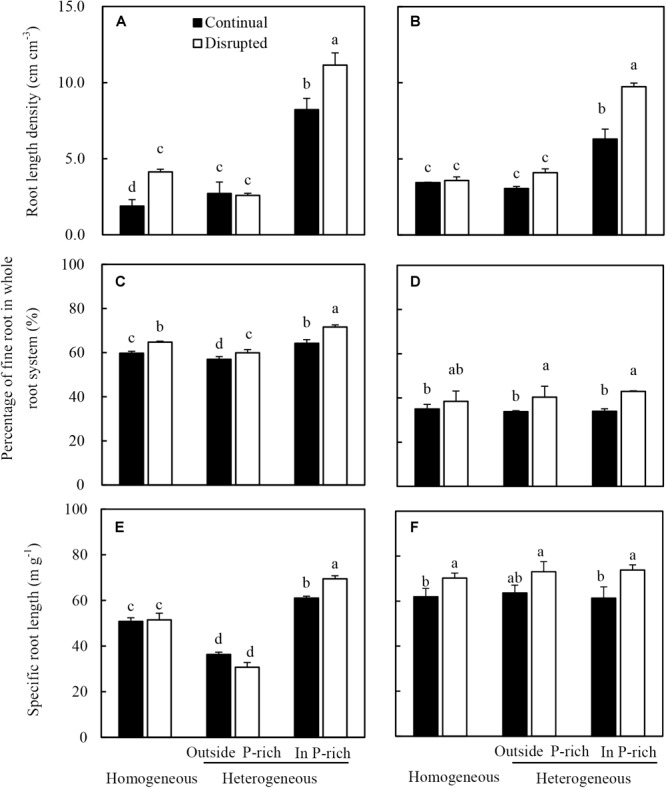
Effect of continual vs disrupted light condition and homogeneous vs. heterogeneous phosphorus (P) supply on root length density (**A**: maize, **B**: soybean), on percentage of fine root in whole root system (**C**: maize, **D**: soybean), and on specific root length (**E**: maize, **F**: soybean). Plants were grown in rhizo-box at two P supply treatments (homogeneous = homogeneous P supply, heterogeneous = heterogeneous P supply) and two light conditions (continual = continual light condition, disrupted = disrupted light condition). Data are average of three replicates and bars represent standard errors. Data with different letters are significantly different (*p* < 0.05). Fine roots were defined as roots with a diameter less than 2 mm.

The root length density of maize in the P-rich zone in the heterogeneous treatment was not only higher than the outside the P-rich zone, but also higher than in the homogeneous P treatment (Figure 4A). Moreover, this increase was more pronounced under disrupted light condition. The fine roots percentage of maize significantly increased under the disrupted light condition irrespective of P supply, particularly in the P-rich zone (Figure 4C). In the P-rich zone, the specific root length of maize significantly enhanced under the disrupted light condition compared to continual light condition (Figure 4E).

The root length density (Figure 4B) of soybean generally followed similar tendencies to that of maize in the light and P treatments. The fine root percentage and specific root length of soybean significantly increased with exposure to disrupted light conditions as opposed to when light conditions were continual, irrespective of P supply (Figure 4D,F). Seemingly, P treatments had no impact on the fine root percentage and specific root length of soybean, with no significant differences noted between the homogeneous P treatment and heterogeneous P treatment (Figure 4D,F).

### The Carboxylate Concentration in Rhizosphere Soil

**FIGURE 5:**
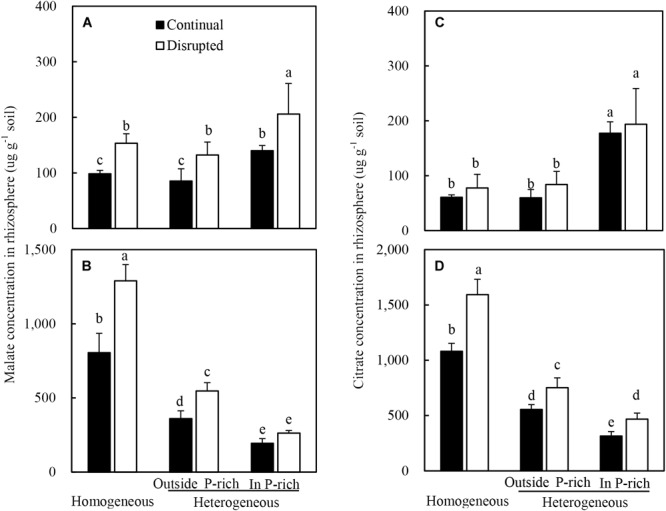
Effect of continual vs disrupted light condition and homogeneous vs. heterogeneous phosphorus (P) supply on rhizosphere soil malate concentration (**A**: maize, **B**: soybean) and on rhizosphere soil citrate concentration (**C**: maize, **D**: soybean). Plants were grown in rhizo-box at two P supply treatments (homogeneous = homogeneous P supply, heterogeneous = heterogeneous P supply) and two light conditions (continual = continual light condition, disrupted = disrupted light condition). Data are average of three replicates and bars represent standard errors. Data with different letters are significantly different (*p* < 0.05).

The root of maize with adequate P supply exuded more malate and citrate than insufficient P supply (Figure 5A,C). Moreover, the malate concentrations, rather than the citrate concentrations, in the rhizosphere soil were elevated substantially under disrupted light condition (Figure 5A,C).

On the contrary, the root of soybean with a low P supply exuded more malate and citrate than adequate P supply (Figure 5B,D). Furthermore, the trend of rhizosphere soil carboxylates concentrations was reversed in soybean as compared to maize, subjected to the same conditions; i.e., under disrupted light conditions citrate levels increased substantially whereas malate concentrations remained constant (Figure 5B,D).

Additionally, the malate and citrate concentrations in the rhizosphere soil of soybean were 10-fold higher than those of maize, particularly in homogeneous P treatment (Figure 5A,B).

### Shoot P Status and Root P Uptake Capacity

**FIGURE 6:**
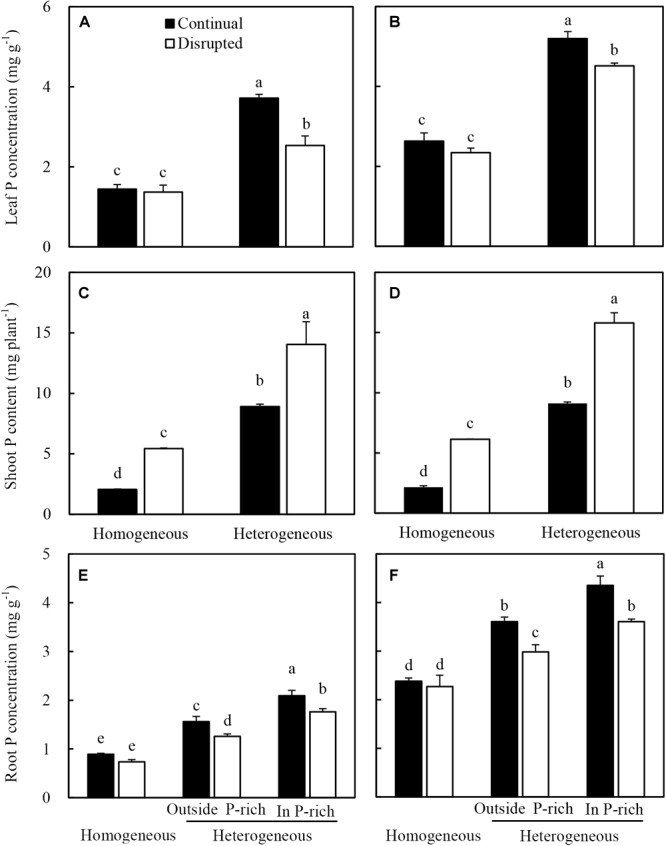
Effect of continual vs. disrupted light condition and homogeneous vs. heterogeneous phosphorus (P) supply on leaf P concentration (**A**: maize, **B**: soybean), on shoot P content (**C**: maize, **D**: soybean), and on root P concentration (**E**: maize, **F**: soybean). Plants were grown in rhizo-box at two P supply treatments (homogeneous = homogeneous P supply, heterogeneous = heterogeneous P supply) and two light conditions (continual = continual light condition, disrupted = disrupted light condition). Data are average of three replicates and bars represent standard errors. Data with different letters are significantly different (*p* < 0.05).

Maize in the heterogeneous P treatment showed higher leaf P concentration than the homogeneous P treatment (Figure 6A). In the heterogeneous P treatment, leaf P concentration of maize decreased under the disrupted light condition compared with the continual light condition. However, leaf P concentrations remained constant in the homogeneous P treatment, irrespective of light conditions (Figure 6A). The heterogeneous P treatment and disrupted light condition caused increased shoot P acquisition of maize (Figure 6C). The root P concentration of maize in the heterogeneous P treatment, particularly in the P-rich zone was greater than that in the homogeneous P treatment as well as outside the P-rich zone (Figure 6E). However, the disrupted light condition, with the exception of the homogeneous P treatment, markedly decreased the root P concentration of maize (Figure 6E).

The leaf P concentration (Figure 6B), shoot P acquisition (Figure 6D) and root P concentration (Figure 6F) of soybean generally followed similar tendencies as maize under the variable light and P treatments.

### Photosynthetic Efficiency and Light Capture of Leaves

**FIGURE 7:**
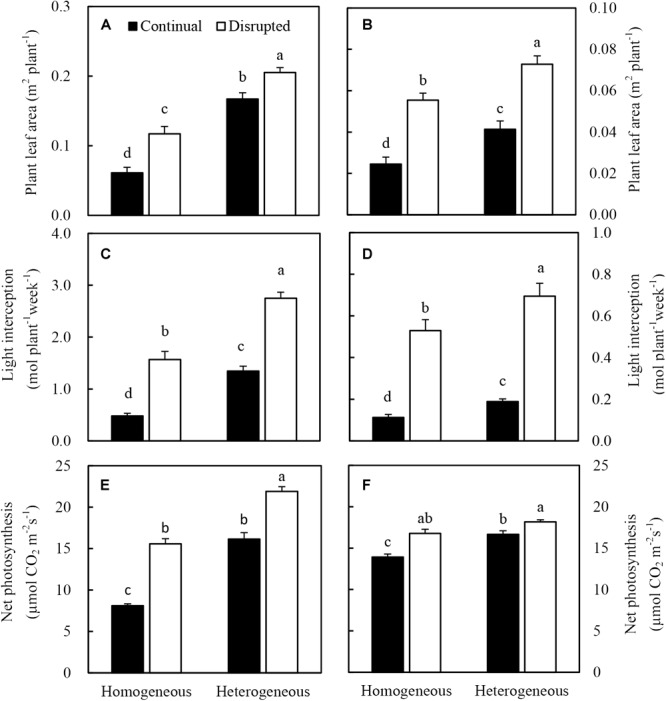
Effect of continual vs disrupted light condition and homogeneous vs. heterogeneous phosphorus (P) supply on plant leaf area (**A**: maize, **B**: soybean), on light interception (**C**: maize, **D**: soybean), and on net photosynthesis rate (**E**: maize, **F**: soybean). Plants were grown in rhizo-box at two P supply treatments (homogeneous = homogeneous P supply, heterogeneous = heterogeneous P supply) and two light conditions (continual = continual light condition, disrupted = disrupted light condition). Data are average of three replicates and bars represent standard errors. Data with different letters are significantly different (*p* < 0.05).

Heterogeneous P supply increased the leaf area of maize, consequently, the light interception of plants increased (Figure 7A,C). Net photosynthesis (Pn) of maize significantly increased under the disrupted light condition as opposed to continual light condition and also enhanced, particularly under heterogeneous P supply (Figure 7E). The increased Pn was correlated with optimized efficiency of light capture, especially under disrupted light conditions (Figure 7C).

The leaf area (Figure 7A), light interception (Figure 7D), and Pn (Figure 7F) of soybean generally followed similar tendencies as maize. However, the Pn was not enhanced by heterogeneous P treatment in disrupted light condition.

### Carbohydrate Accumulation in Leaves and Roots

**FIGURE 8:**
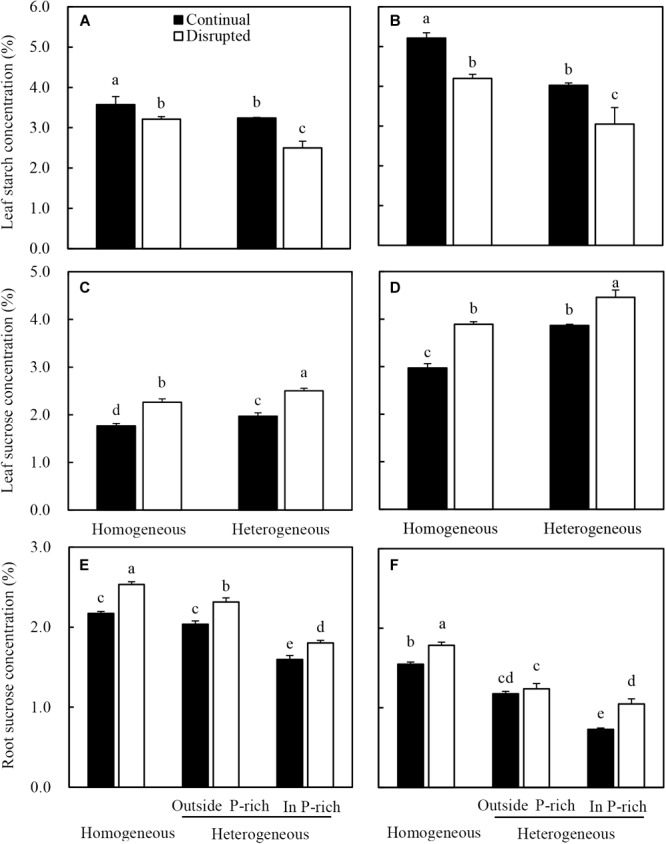
Effect of continual vs. disrupted light condition and homogeneous vs. heterogeneous phosphorus (P) supply on leaf starch concentration (**A**: maize, **B**: soybean), on leaf sucrose concentration (**C**: maize, **D**: soybean), and on root sucrose concentration (**E**: maize, **F**: soybean). Plants were grown in rhizo-box at two P supply treatments (homogeneous = homogeneous P supply, heterogeneous = heterogeneous P supply) and two light conditions (continual = continual light condition, disrupted = disrupted light condition). Data are average of three replicates and bars represent standard errors. Data with different letters are significantly different (*p* < 0.05).

Leaf starch concentrations of maize under the continual light condition were higher than those under the disrupted light condition, whilst it decreased in the heterogeneous P treatment compared with homogeneous P treatment (Figure 8A). The sucrose concentrations in leaves and roots of maize increased under disrupted light conditions and were also affected by P treatments, with elevated levels under heterogeneous P supply (Figure 8C,E). Contrastingly, sucrose concentration in the root of maize in homogeneous P treatment was relatively higher than those of roots either outside or inside the P-rich zone in the heterogeneous P treatment (Figure 8E).

Similar trends were found for soybean leaf starch (Figure 8B), leaf sucrose (Figure 8D) and root sucrose concentration (Figure 8F) under the same light and P conditions.

### Relationships Between Fine Root Percentage, Rhizosphere Soil Malate Concentration, and Phosphorus Concentration in Leaves, or Sucrose Concentration in Roots

**FIGURE 9:**
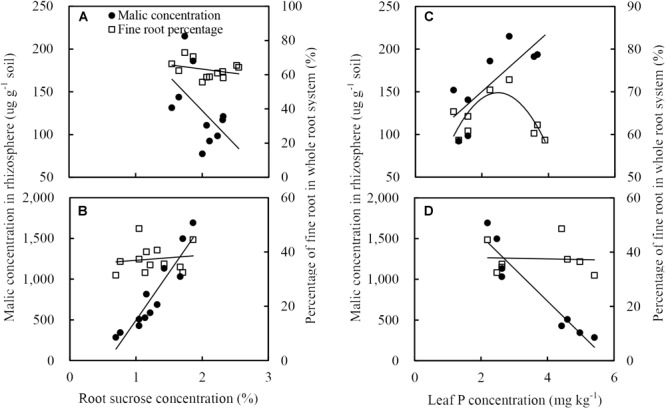
Relationships between soil malate concentration in rhizosphere (black circles) and fine root percentage (open squares), and sucrose concentration in roots (**A**: maize, **B**: soybean), or phosphorus concentration in leaves (**C**: maize, **D**: soybean). There was only showed the relationships between malate concentration in the rhziosphere and sucrose eoncentration in roots, or phosphorus concentration in leaves, because the citrate concentration in rhizosphere soil generally followed similar tendencies to malate.

The fine root percentage of maize did not positively correlate to root sucrose concentration (Figure 9A) but positively related to the leaf P concentration (Figure 9C). The malate concentration in the rhizosphere soil of maize was inversely related to the root sucrose concentration (Figure 9A) and positively related to the P concentration in leaf (Figure 9C).

The malate concentration in the rhizosphere soil of soybean was positively related to the root sucrose (Figure 9B) and inversely related to the leaf P concentration (Figure 9D). In addition, neither root sucrose (Figure 9B) nor leaf P concentration (Figure 9D) was correlated to the fine root percentage.

## Discussion

### The Light Capture of Maize and Soybean Increased Under the Disrupted Light Condition

This study was conducted to investigate whether a change in planting pattern in a low light intensity area could increase light capture in leaves, which could possibly augment photosynthesis. In conjunction with how localized P supply could affect the concentration of P and sucrose in plant tissue which increased the P uptake capacity of maize and soybean by regulating root growth.

Wide-narrow row distance planting patterns are widely used by farmers in China. It is a typical system for the region with more than 2.8 × 10^7^ ha of annual crops being intercropped in China (Li et al., 2007). Many studies reported that the increased light capture in border rows resulted in greater yields in strip intercropping than those in mono-cropping (Zhang et al., 2008, 2015; Zhu et al., 2016; Gou et al., 2017; Wang et al., 2017). The light capture of plants in maize/soybean relay strip intercropping was also higher than that of the corresponding mono-crops in southwest China with low solar radiation (Gao, 2015; Chen et al., 2016). The observations in this study that the light capture of maize and soybean under the disrupted light condition was higher than those under the continual light condition (Figure 7C,D) corroborated results from previous studies. Theoretically, crops in wide-narrow row distance planting patterns obtain a low amount of light capture because half of the leaves on the narrow side are normally shaded by neighboring plants. However, plants showed high phenotypic plasticity to complement light capture in plant mixtures. In a maize/wheat intercropping system, light capture was 23% higher in the maize/wheat system than that of the observed value for monocultures, with 64% of the increase attributable to phenotypic plasticity (Zhu et al., 2015). In the present study, soybean showed high phenotypic plasticity, such as leaves aggregated on one side of non-adjacent plants under the disrupted light condition scenario (Supplementary Figure S2B), which might has contributed much to the improved light capture by this crop. Additionally, leaf numbers, single leaf area, and, consequently, total leaf area increased also contributed to the improved light capture under disrupted light condition (Supplementary Figures S3A–D and Figure 7A,B). Low light and low P aggravated senescence of leaves situated lower down on the main stem, which resulted in a decrease in leaf numbers and area (Lim et al., 2007; Sakuraba et al., 2014) (Supplementary Figures S1D, S2A,C,D). These results provided evidence in favor of the hypothesis that changes in the planting pattern of maize and soybean from equal-width row distance to narrow-width row distance resulted in improving light capture of crops in low light intensity areas.

### Root Morphological Responds to Localized Soil P Supply and Light Condition

To capture more nutrients, root morphological of many species exhibited high plasticity to the localized supply of nutrients (Bilbrough and Caldwell, 1995; Hodge et al., 2000; He et al., 2003; Li H.B. et al., 2014). This also held true for maize in this study, where, heterogeneous supply of P induced root proliferation in the P-rich zone and increased plant P acquisition (Jing et al., 2012; Li et al., 2012; Ma et al., 2013; Zhang et al., 2015). The data reported here were consistent with the observations that P uptake, root length density, and consequently, root dry weight density of maize and soybean increased in the P-rich zone with localized P supply (Figure 3C,D, 4A,B, and Supplementary Figure S4). However, the mechanisms of P-dependent changes in root proliferation in response to local P supply were not fully understood (Hodge, 2004; Shen et al., 2011). Generally, root growth was initiated by the signal of low leaf P and high root sucrose concentration (Shane et al., 2003; Hill et al., 2006; Teng et al., 2013). In the present study, the increasing leaf light interception augmented the response of maize and soybean root to the heterogeneous P supply. In heterogeneous P treatment, increased light capture decreased the leaf P concentration of maize and soybean (Figure 6A,B), which might be due to increased photosynthesis and shoot growth rate exceeding the P supply ability of roots to leaf, such as high net Pn and sucrose concentration in leaves under disrupted light condition (Figure 7A,B, 8C,D). Moreover, the root dry weight density and root length density of maize and soybean enhanced under disrupted light condition (Figure 3C,D, 4A,B). Thus, high light may cause growth-induced P starvation in the shoot that produces a systemic signal to induce root growth. These results are consistent with the previous studies on rice and lupine with high light intensity, which induced root growth (Wissuwa et al., 2005; Cheng et al., 2014). In addition, the leaf P concentrations of maize and soybean in homogeneous P treatment were twofold lower than in heterogeneous P treatment (Figure 6A,B), but root growth was severely inhibited under homogeneous P supply (Figure 3C,D). One possible explanation may be that plant leaf growth under P-deficiency decreased significantly and, subsequently inhibited root growth (Mollier and Pellerin, 1999; Plénet et al., 2000). Therefore, these results provided evidence that favored hypothesis i. In the heterogeneous P treatment, maize and soybean roots presented more sucrose under disrupted light, compared with continual light, resulting relatively great root weight density and root length density (Figure 3C,D, 4A,B). The same results reported previously that the proliferation of roots followed the stimulation of carbohydrate allocation to roots (Watt and Evans, 1999; Hammond and White, 2008; Postma et al., 2014). The sucrose concentrations of maize and soybean roots in the homogeneous P treatment were higher than those in the P-rich zone in heterogeneous P treatment, but the root growth was severely inhibited compared to in the enriched P patch (Figure 3C,D, 4A,B), which might provide evidence to reject hypothesis B. One possible explanation might be that reductions of root growth under P deficiency were not caused by source limitations but were more due to the direct negative effect of low P availability (Wissuwa et al., 2005). The results of root P concentration in the homogeneous and heterogeneous P treatments here were consistent with this view (Figure 6E,F).

Furthermore, the localized P supply inducing root proliferation was not only regulated by the signal of leaf P but also root sucrose concentration. Such as in the root-split plants in heterogeneous P treatment, the root growth outside the P-rich zone was lower than the P-rich zone (Figure 3C,D, 4A,B), although the root outside the P-rich zone showed higher sucrose concentration (Figure 8E,F). The root proliferation in a nutrient patch depended on the relative concentration of nutrients as it pertains to the rhizosphere vs. bulk soil (Hodge, 2004; Shen et al., 2005; Giehl et al., 2013), and which may also responsive to phytohormone synthesis and transport. For example, ethylene is known to be involved in the regulation of decreasing root gravitropic response under P limitation to increase root proliferation in the P-rich zone (Bonser et al., 1996; Lynch and Brown, 2008). Furthermore, the basipetal flow of auxin was regulated by light intensity which facilitates the root proliferation in P -rich patches (Bhalerao et al., 2002; Costigan et al., 2011).

Localized P supply only increased the fine root percentage and specific root length of maize rather than soybean. Previous studies reported that a localized nutrient (P and nitrogen) supply increased the production of maize fine roots (diameter <0.2 mm) (Jing et al., 2010; Li H.B. et al., 2014; Wen et al., 2017). Root morphology in response to heterogeneous nutrient supply was influenced by the root diameter. Therefore, fine-rooted species are more responsive to nutrient-rich patches than species with coarse roots (Fitter, 1994; Hodge, 2004). Because plants with fine roots have high specific root length per unit carbon, the metabolic demand per unit of root length in the root system decreased, and therefore, soil P exploration and acquisition increased at a minimal energy cost (Lambers et al., 2006; Zobel et al., 2007; Pang et al., 2009; Lynch, 2015). In this study, most maize roots had a diameter less than 0.2 mm, whereas the root diameter of soybean was more than 0.2 mm, indicating that maize had finer roots than soybean (Figure 4C,D). Thus, maize rather than soybean, responded to localized P supply by altering the root architecture for a better interception, as previously reported by Lyu et al. (2016). Similar results also showed that species of Graminaceae (maize and wheat), but not species of Leguminosae (faba bean and chickpea), responded to localized P supply by altering the root architecture for better interception (Li H.B. et al., 2014). A positive relationship was observed between leaf P concentration and fine root percentage of maize, which agreed with the view of the production of fine root in maize affected by leaf P concentration (Figure 9C) (Wen et al., 2017). However, the fine root percentage of soybean did not significantly respond to root sucrose (Figure 9B) and leaf P concentration (Figure 9D), which might be ascribed to soybean response to soil P condition by altering root physiology rather than root morphology (Lyu et al., 2016).

### Root Physiological Responds to Light Condition and Localized Soil P Supply

More light capture increased malate and citrate concentration in the rhizosphere of maize and soybean, even under localized P application condition (Figure 5). This was consistent with the results obtained in white lupin under high light intensity (Cheng et al., 2014). In this experiment, root sucrose concentration under disrupted light conditions was much higher than continual light conditions, whilst the malate concentration in soybean and maize rhizosphere was positively responded to root sucrose (Figure 9A,B). These observations were consistent with the results that root sucrose concentration regulated malate and citrate exudation of maize and soybean, and give credence to findings from a previous study pertaining to citrate exudation of white lupin (Cheng et al., 2014). Other systemic signals could also contribute to the exudation of carboxylates instead of the high light intensity increased carboxylate exudation by translocation of sucrose to roots. This may include low leaf P concentration, which has been found to stimulate root carboxylate exudation (Shen et al., 2005; Li et al., 2008).

However, in this study low leaf P concentration did not stimulate carboxylate exudation in maize root, as reported previously that the increased carboxylate exudation in the rhizosphere of maize was induced by high leaf P concentration (Corrales et al., 2007; Zhang et al., 2015; Liu et al., 2016; Lyu et al., 2016). Localized P supply coincided with increases in the malate and citrate concentrations in the rhizosphere of maize, since external P supply increased the P in leaves (Figure 5A,C). This was in agreement with another report on the same species (Wen et al., 2017). Maize showed opposite root carboxylate exudation tendencies to Leguminoseae species regulated by leaf P status (Li H.B. et al., 2014; Lyu et al., 2016). One possible explanation might be that maize responded to P-insufficiency by altering root morphology rather than increasing root exudation, because fibrous roots (e.g., maize) respond to variable P supply through expanding the root surface area to increase the absorption of available P spatially (Lyu et al., 2016; Wen et al., 2017). The carboxylate exudation of maize was markedly lower than that of Leguminoseae species (Pearse et al., 2006, 2007; Rose et al., 2010; Li L. et al., 2014), which provided additional evidence to support that maize responds to P starvation by changing root morphology rather than root physiology.

P deficiency in leaves significantly stimulated the malate and citrate exudation in the rhizosphere of soybean in homogeneous P treatment. In contrast to maize, localized P supply significantly increased the P concentration of leaves and decreased the carboxylate concentration in the rhizosphere of soybean seedlings (Figure 5B,D). In addition, a negative relationship was observed between leaf P concentration and malate concentration in the rhizosphere (Figure 9D). These results suggested an important influence of localized P supply on soybean root malate and citrate exudation by affecting the leaf P status, which was in agreement with lupin under localized P supply experiment (Shen et al., 2005). It also corresponded well with the observations that leaf P regulated root carboxylate exudation in Leguminoseae species (e.g., faba bean: Li L. et al., 2014, Zhang et al., 2015; white lupine and chickpea: Li L. et al., 2014; Lyu et al., 2016). However, malate and citrate concentration in the outside P-rich zone was higher than in the P-rich zone, which coincided with the observation that the root P outside the enriched P-patch part was lower than in the P-rich zone (Figure 6F), indicating a clear effect of the localized P supply on carboxylate exudation.

## Conclusion

Our results demonstrated that the light capture of maize and soybean under disrupted light conditions were higher than those under continual light conditions in a low solar radiation area. It means that altering planting patterns of crops from equal-width row distance to narrow-width row distance is a useful management strategy to facilitate better light interception, particularly under suboptimal light conditions in low solar radiation areas. The increasing light interception and localized P supply increased maize root proliferation, root morphological plasticity (high fine root percentage, high specific root length) and P uptake capacity. The increased light interception and localized P supply also promoted soybean root growth, but the P supply treatment did not affect the root morphology. However, for soybean, the increased light interception enhanced malate and citrate exudation, which were also induced by concomitant P deficient conditions. The light and P are possibly integrated by maize and soybean through both the P and sucrose concentrations in leaves and roots to determine plant growth. This study provided new insights into the P uptake capacity of maize and soybean in the “wide-narrow row distance planting pattern,” which are important for increasing crop productivity and P fertilizer use efficiency through optimizing planting patterns and soil P supply strategies in low solar radiation areas.

## Author Contributions

TZ, LW, WL, and WY carried out the design of this research work and writing this manuscript. TZ, LW, and YD carried out the plant cultivation, chemical analysis and statistical analysis of this work. LZ, YG, and SL participated in experiment management.

## Conflict of Interest Statement

The authors declare that the research was conducted in the absence of any commercial or financial relationships that could be construed as a potential conflict of interest.

## Supplementary Material

FIGURE S1 The picture showed “wide-narrow row distance planting patterns” of maize **(A)** and soybean **(B)** in the field under low solar radiation area of southwest of China. The picture of **(D)** showed the down leaves of soybean were dropped because of low light interception in continual light condition, but the leaves of soybean keep a longer green period in disrupted light condition **(C)**. The picture of **(E)** showed the soybean was harvested in disrupted light condition (left) and in continual light condition (right).

FIGURE S2 The picture showed maize **(A)** and soybean **(B)** in continual vs. disrupted light condition and homogeneous vs. heterogeneous phosphorus (P) supply treatment. The soybean showed high phenotypic plasticity **(C,D)**. HomC, homogeneous P supply and continual light condition. HomD, homogeneous P supply and disrupted light condition. HetC, heterogeneous P supply and continual light condition. HetD, heterogeneous P supply and disrupted light condition.

FIGURE S3 Effect of continual vs. disrupted light condition and homogeneous vs. heterogeneous phosphorus (P) supply on the number of leaves (**A**: maize, **B**: soybean), and on single leaf area (**C**: maize, **D**: soybean). Plants were grown in rhizo-box at two P supply treatments (homogeneous = homogeneous P supply, heterogeneous = heterogeneous P supply) and two light conditions (continual = continual light condition, disrupted = disrupted light condition). Data are average of three replicates and bars represent standard errors. Data with different letters are significantly different (*p* < 0.05).

FIGURE S4 Effect of continual vs. disrupted light condition and homogeneous vs. heterogeneous phosphorus (P) supply on root P content (**A**: maize, **B**: soybean). Plants were grown in rhizo-box at two P supply treatments (homogeneous = homogeneous P supply, heterogeneous = heterogeneous P supply) and two light conditions (continual = continual light condition, disrupted = disrupted light condition). Data are average of three replicates and bars represent standard errors. Data with different letters are significantly different (*p* < 0.05).
