# Supplementary material for: Interactions Between Light Intensity and Phosphorus Nutrition Affect the P Uptake Capacity of Maize and Soybean Seedling in a Low Light Intensity Area
Source: Front Plant Sci. 2019 Feb 19;10:183. doi: 10.3389/fpls.2019.00183 (PMC6390497; doi:10.3389/fpls.2019.00183)
Supplement: FIGURE S1 — The picture showed “wide-narrow row distance planting patterns” of maize (A) and soybean (B) in the field under low solar radiation area of southwest of China. The picture of (D) showed the down leaves of soybean were dropped because of low light interception in continual light condition, but the leaves of soybean keep a longer green period in disrupted light condition (C). The picture of (E) showed the soybean was harvested in disrupted light condition (left) and in continual light condition (right). [file Table_1.DOCX]

**Fig.** S**1**

**A**

**Maize**


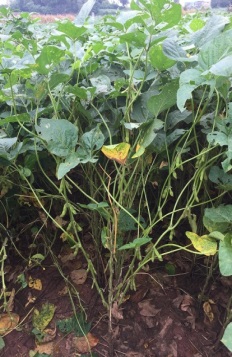

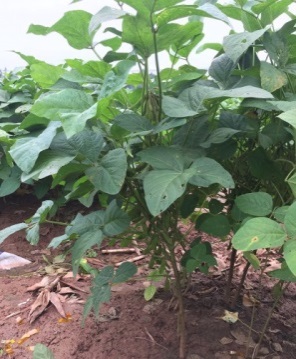

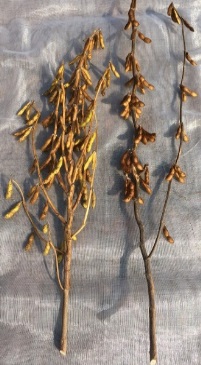

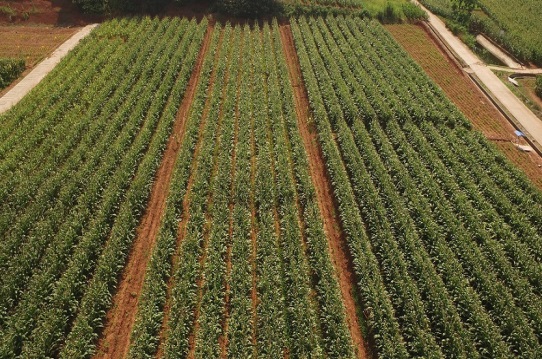

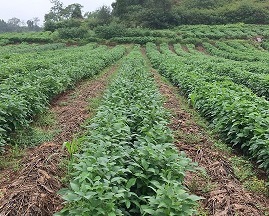


**Right**

**Left**

**Narrow row**

**Wide row**

**E**

**D**

**C**

**Soybean**

**B**

**Fig. S2**

**HetD**

**HotD**

**HetC**


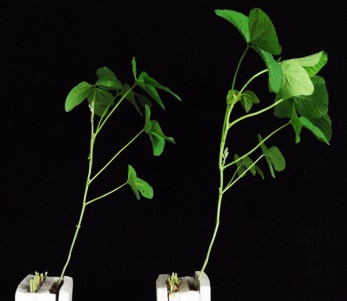

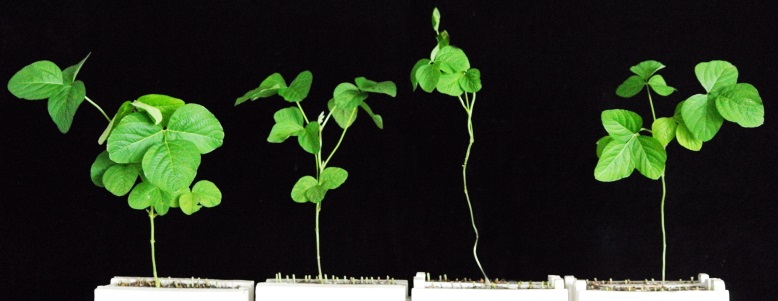

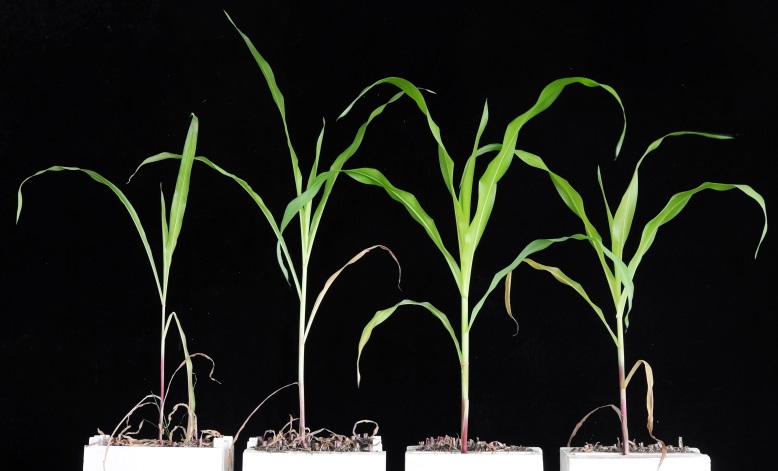

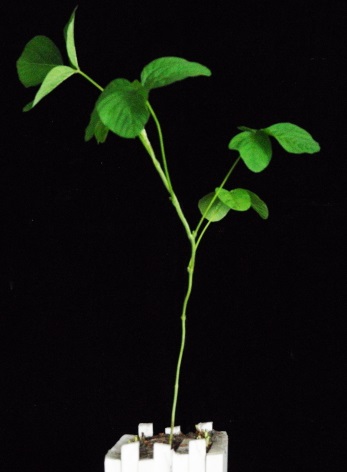


**HetD**

**HotD**

**HotC**

**Neigh. Plants**

**A**

**B**

**HetC**

**HotD**

**HetD**

**HotC**

**HetC**

**D**

**C**

**Neigh. Plants**

**Fig. S3**

**C**

**A**

Number of leaves (number Plant^-1^)

The area of single leaf (cm^2^ per leaf)

**D**

**B**

**Fig. S4**

Root P content (mg plant^-1^)

**A**

**B**

Homogeneous

Heterogeneous

Outside P-rich P-rich

In P-rich
